# Case Report: Intraoperative radiotherapy as the new standard of care for breast cancer patients with disabling health conditions or impairments

**DOI:** 10.3389/fonc.2023.1156619

**Published:** 2023-05-18

**Authors:** Michael Omosule, Shiroma De Silva-Minor, Nathan Coombs

**Affiliations:** ^1^ GKT School of Medical Education, King’s College London, London, United Kingdom; ^2^ Department of Clinical Oncology, Oxford University Hospitals NHS Foundation Trust, Oxford, United Kingdom; ^3^ Department of Breast Surgery, Great Western Hospitals NHS Foundation Trust, Great Western Hospital, Swindon, United Kingdom

**Keywords:** intraoperative radiotherapy, breast cancer, radiotherapy, TARGIT, IORT, quality of life, breast

## Abstract

In selected patients, intraoperative radiotherapy (IORT) offers an alternative to standard external beam radiotherapy (EBRT) while providing equivalent breast cancer control outcomes. After IORT, most patients do not require external beam radiotherapy and thus avoid the need to travel to and from a radiotherapy centre in the weeks after surgery. EBRT is associated with an increased risk of non-breast cancer mortality and poorer cosmetic outcomes while increasing patient travel time, emissions associated with travel and time spent in the hospital. Consequently, EBRT is associated with an overall reduction in quality of life compared to IORT. Patients with other on-going health conditions or clinical impairments are likely to be affected by the daily radiotherapy requirement. Should these patients be consulted during their pre-operative assessment as to options to undergo IORT? This paper describes a case of IORT and follow up in a functionally blind patient. Quality of life effects are elucidated and further support the use of IORT in selected breast cancer patients with health conditions or impairments.

## Introduction

Breast cancer is the most common cancer, accounting for 15% of all cancers in the United Kingdom. Approximately 56,000 new cases of breast cancer are diagnosed annually (1). Breast cancer incidence increases with age, with 80% of new diagnoses occurring in women aged 50 years or older (2). Treatment is largely determined by the patient’s health, menopause status, tumour size, nodal status and evidence of any metastatic disease. With high screening rates in the UK, most breast cancers are discovered at an early stage and 80% will be treated with breast conserving surgery, by wide local excision or mastectomy. Adjuvant whole breast external beam radiotherapy (EBRT) is delivered to 80% of patients post lumpectomy to improve tumour control and reduce mortality (1, 3).

Adjuvant radiotherapy is a valuable component of breast cancer therapy in those receiving breast conserving treatments. At present in the UK, EBRT is delivered with a five-fraction regime as the standard of care. However, this may come with considerable physical, psychological and financial consequences (3–5). After breast conserving surgery, 80% of patients need to travel daily to radiotherapy centres, to receive at least five treatments. Other longer regimes extending over several weeks might be necessary (6, 7). Following the trauma of surgery, travelling to and from radiotherapy centres can be physically challenging for some, especially given that many breast cancer patients are elderly and have comorbidities (8, 9). Alongside this, daily travel can incur a significant monetary charge if travelling long distances. A large financial and time burden is placed upon many, as two thirds of breast cancer patients live over 13 miles away from their nearest radiotherapy centre. Accompanying this significant travel is the environmental impact of travel for cancer treatments (7, 10).

As an alternative, the targeted intraoperative radiotherapy (TARGIT-A) trial has demonstrated that intraoperative radiotherapy (IORT) can deliver non inferior treatment outcomes compared to EBRT for eligible patients (7, 10). Furthermore, IORT significantly reduces the rate of non-breast cancer mortality and eliminates the need for external beam radiation therapy in 80% of patients. Patients who receive IORT have a better quality of life (QOL) and have a reduced financial and time burden post lumpectomy. Reducing the pressure on existing radiotherapy departments through the use of IORT would further reduce strain placed on the NHS and may reduce spending (7, 11). This paper highlights the benefits of using IORT to treat elderly breast cancer patients and those with comorbidities.

## Case

**Figure 1:**
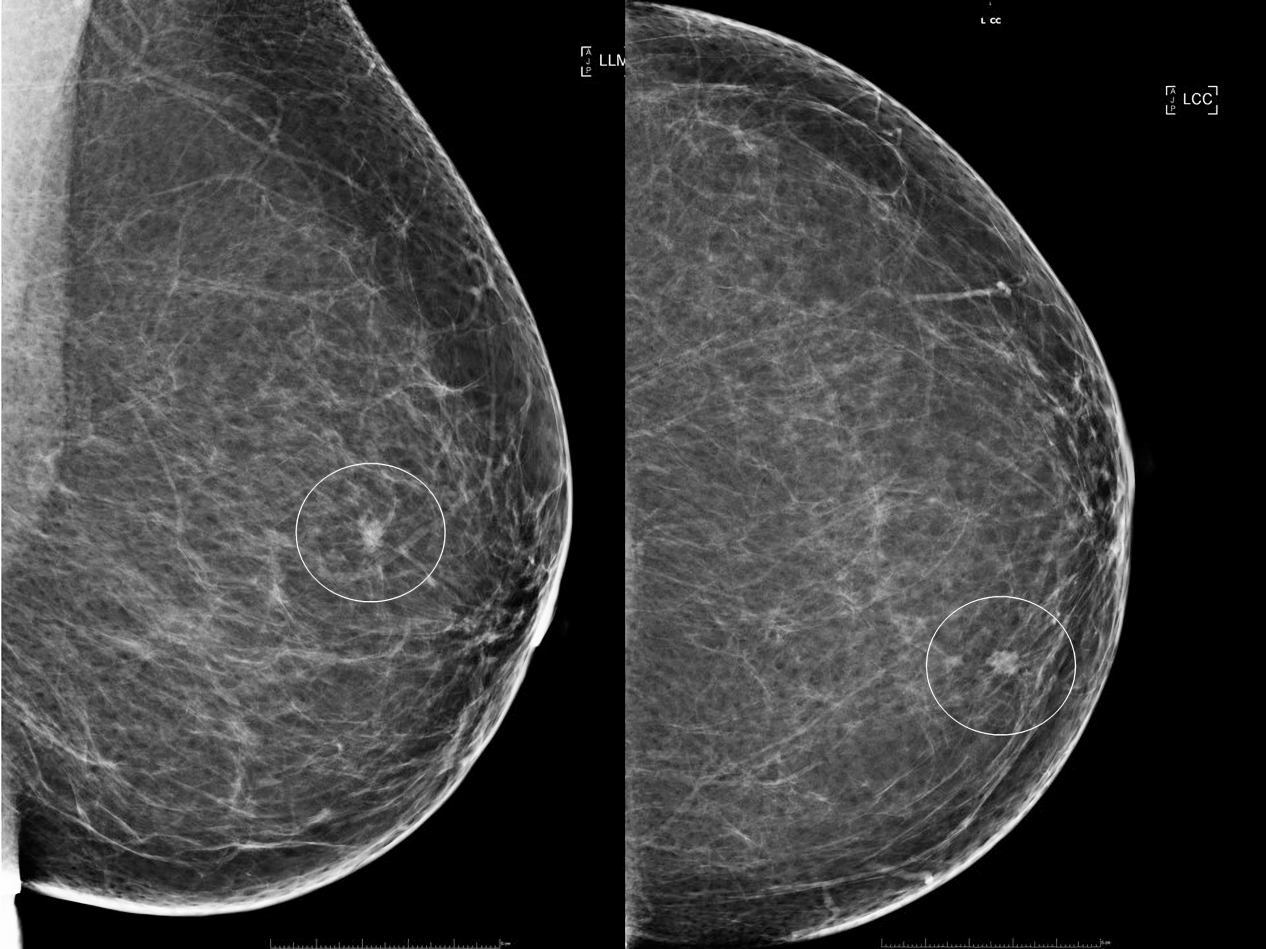
Plain x-ray mammograms demonstrating a small spiculated mass (circled) within the upper inner segment of the breast. Left image: Mediolateral oblique view. Right image: Craniocaudal view.

A 64-year-old female presented with a small mass in her left breast during her mammography screening appointment. The mass within the upper inner quadrant of the left breast was irregular, spiculated and with calcifications (**Figure 1**). Subsequently, breast ultrasound confirmed the presence of a mass, but with no obvious enlargement of the axillary nodes. Ultrasound guided core biopsy demonstrated a grade 2, hormone positive [ER+ve 280/300, PR+ve 300/300], HER2-ve invasive ductal carcinoma with no ductal carcinoma *in situ* (DCIS). The patient had a previous diagnosis of a right breast cancer treated in another breast centre, with a mastectomy as well as reconstruction and axillary clearance for DCIS (with no invasion) 13 years earlier. The patient had long-standing significant lymphedema of her right arm as well as a past medical history of cervical spondylosis, osteoarthritis of the carpo-metacarpal joints, distal interphalangeal and proximal interphalangeal joints of the hands, and fibromyalgia. She also had a history of Meige syndrome, characterised by involuntary and often forceful contractions of the muscles around the eyes, jaw and tongue and tearing which caused a functional blindness. There was no family history of breast disease.

The risks and benefits of four treatment options were discussed with the patient:

1. A wide local excision and sentinel node biopsy followed by adjuvant external beam whole breast radiotherapy for 5 days a week over three weeks. (This was the EBRT standard of care at the time of diagnosis)
2. A wide local excision and sentinel node biopsy with the omission of radiotherapy. (Though strictly speaking, the evidence for omission of adjuvant radiotherapy is in patients 65 years and older and required patients to be compliant with 5 years of adjuvant endocrine therapy. Given the various comorbidities there was a high likelihood that this lady would not be able to tolerate the side effects of endocrine therapy, particularly the musculoskeletal side effects associated with Aromatase Inhibitors. A treatment plan including radiotherapy should be prioritised in case the patient cannot tolerate the side effects.)
3. Consideration of neoadjuvant endocrine treatment for a short period of time whilst NHS funding for IORT was being sought, after which a wide local excision and delivery of IORT would take place. (Again, there were concerns about tolerability of endocrine therapy side effects.)
4. A wide local excision with self-funded IORT. The patient was made aware that a fifth of patients who receive IORT may be treated with adjuvant EBRT depending on the histology findings.

After discussion with the patient and the multidisciplinary team, it was decided that she would be a good candidate for a wide local excision with IORT. Sight difficulties were the major factor that influenced her decision. EBRT would have required her to travel 40 minutes each way by car, and 3 hours each way by public transport. The guilt of putting pressure on her disabled husband to assist with daily travel for three weeks was immense, and the thought of having nothing further to face after the surgery brought relief. The patient’s daughter chose to fund her treatment to allow surgery and IORT in a timely manner.

**Figure 2:**
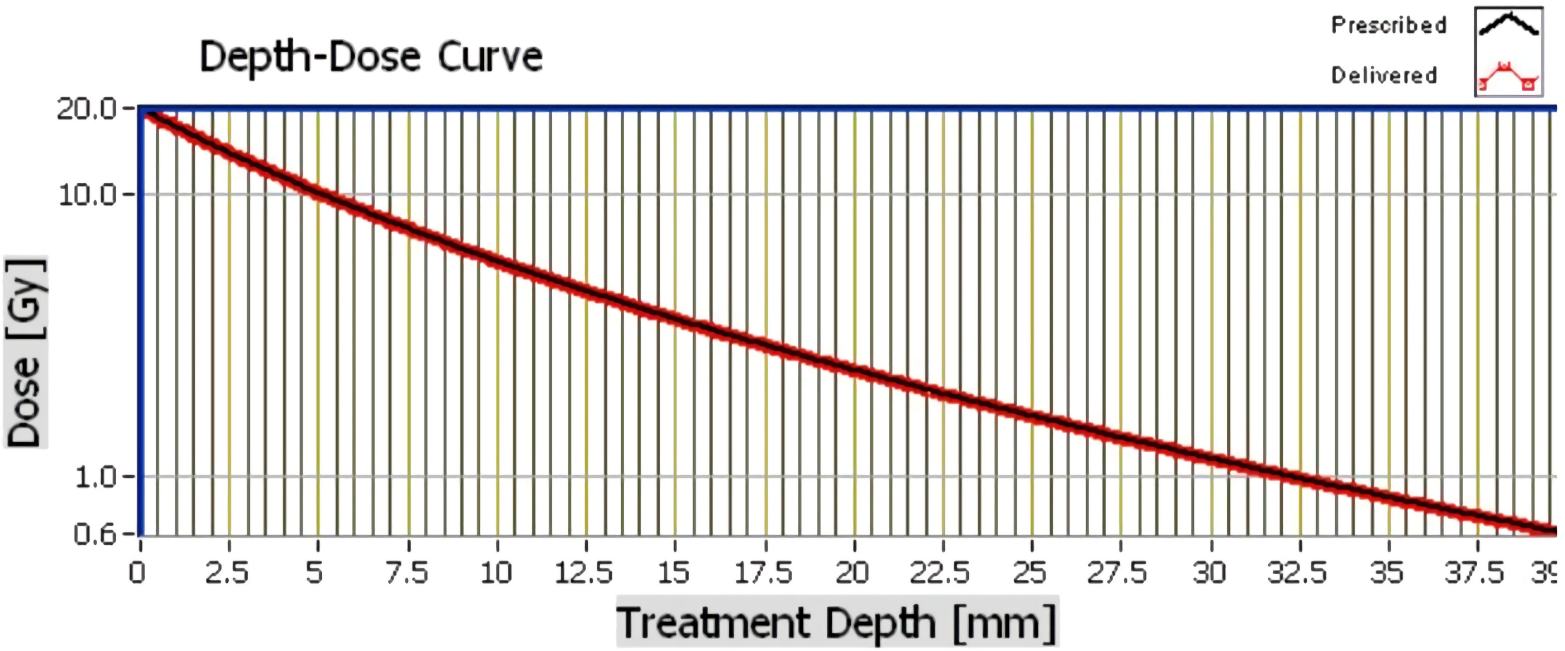
Radiotherapy depth dose curve for intra-operative radiotherapy delivery.

Intra-operative radiotherapy was delivered immediately after wide local excision using a miniature electron beam driven X-ray source (Intrabeam™ (Carl Zeiss Meditec, Oberkochen, Germany)) (12). Twenty gray of radiation was delivered from the surface of a 4cm spherical applicator directly to the excision cavity for 27 minutes (**Figure 2**). The patient chose to stay overnight for observation. Recovery was uneventful with no complications. Final histology reported a 7mm, grade 1, hormone receptor positive tumour resected with microscopically clear margins. Sentinel node biopsy was free from tumour. The patient was commenced on a 5-year course of an aromatase inhibitor, anastrozole, along with Vitamin D and B12 supplements, which immediately caused distressing muscular and joint pain in upper and lower limbs, polydipsia, polyurea, hot flushes and tiredness. After discussion, the patient agreed to take 2-4 week anastrozole breaks to relieve side effects and extend the duration to 10 years. Since then, the patient has remained stable having only taken three, two-week breaks over the seven-year period. Annual blood tests and bone density scan results have remained within normal ranges and annual surveillance mammography has shown no signs of recurrence for seven years.

## Discussion

Post-surgical EBRT has been described as fatigue inducing, possibly owing to its daily radiotherapy requirements. Symptoms are exacerbated in comorbid and physically impaired groups (3, 13–15). Moreover, Muszalik et al. (16) reports that the 61-70 age group suffer the worst fatigue symptoms. Given that many breast cancer patients undertaking radiotherapy are older and have co-morbidities, efforts should be made to relieve stressors and prioritise QOL. The TARGIT-A trial, undertaken by Vaidya et al. (7) showed that targeted intraoperative radiotherapy can prevent the need for adjuvant EBRT in 80% ofpatients. This is done without compromising patient safety or increasing the rate of disease occurrence (7).

Other methods of delivering IORT have been demonstrated, including that described in the ELIOT trial. On the contrary, the ELIOT trial demonstrated that IORT was inferior to EBRT (17). The difference in efficacy may be explained by the difference in radiation type, applicator or methodology. The ELIOT method employs a linear electron accelerator to deploy radiation in an anterior to posterior manner. IORT delivered in the TARGIT trial uses a probe that contacts the excised tumour bed (18, 19).

Over the last 30 years there has been little improvement in breast cancer survival in patients with severe comorbidities. Furthermore, breast cancer mortality is increased by the severity and number of comorbidities even after adjusting for age and stage and these may be as important as cancer stage in predicting survival (20). Given that 65% of breast cancer patients have co-morbidities, clinicians should consider that the use of IORT negates the need for EBRT and may reduce the exacerbation of these comorbidities. Thirty-two percent of patients suffer with arthritis and a quarter suffer with cardiovascular disease. These conditions are commonly aggravated by the stress and the travel needed for EBRT (21–23). The x-rays delivered by the Intrabeam™ system have a steep dose gradient, thus only the tissues to a depth of 3cm, directly surrounding the excision site are irradiated. The therapeutic depth however is 6mm. Unnecessary collateral irradiation to the nearby chest wall, heart and lungs is therefore reduced significantly (24). This may explain the published data that demonstrates a significant reduction in non-breast cancer mortality (7).

Moreover, patients are more likely to report poor emotional health and QOL during the course of EBRT (21). A prospective cohort study of women over 65 with a diagnosis of early-stage breast cancer demonstrated that poor health related QOL is directly detrimental to survival, independent of breast cancer prognostic variables (25). Physical function, mental health and social support are three domains that constitute health related QOL, and all are negatively affected by the radiotherapy (21, 25–27). In addition, 90% of patients have reported fatigue as a side effect of radiotherapy, with 30% describing it as severe to intolerable. Schnur et al. (3) captured the thoughts of these patients, with some describing their fatigue as, “totally exhausted to the point I could hardly move”, and “total shutdown”. Mental health in breast cancer radiotherapy patients is also largely affected, with 31% of patients experiencing moderate to severe levels of negative affect and two-fifths experiencing anxiety. Statements such as, “I’m giving in to imagined or real side effects of radiation” and, “I should be finished with crying” were expressed by patients (3, 28). Lack of perceived social support from families, co-workers, bosses and friends is reported by 40% of patients undergoing breast radiotherapy (3). Statements such as, “My co-worker doesn’t seem to understand my need to rush out of work for my treatment”, and “I should have stayed in my abusive marriage because I would not be alone”, highlight the severity of the social issues that some patients experience (3, 28). IORT as a sole treatment in 80% of patients can prevent the exacerbation of these poor quality of life outcomes and should therefore be considered in eligible patients with comorbidities, mental health and those with poor perceived social support (7).

One in five people aged 70 or over are visually impaired. Vision loss is a factor that impairs radiotherapy access (29). In 2013 approximately 1.99 million people in the UK suffered with sight loss or blindness. The prevalence of sight loss has increased by 7.5% in the last decade. This proportion is set to increase further with demographic ageing. The cost of blindness affects patients significantly and restricts their ability to travel independently (30, 31). The travel requirements for EBRT place a burden on visually impaired patients, their families, and their support networks. Patient’s sight and their ability to access safe travel, and support should be seriously considered when determining appropriate radiotherapy (31).

There are numerous advantages with the use of the IORT Intrabeam™ system. Being portable, it can easily be used in most operating rooms within a hospital. IORT is intended as a single-dose treatment and adds about 30 minutes to operative time. This is less than the total time undertaking EBRT radiotherapy. Although EBRT may take only 5-10 minutes to deliver each fraction, the preparation and appointment times often allow 20 minutes (32). Therefore, each patient spends over 100 minutes receiving EBRT. This ignores the time taken for travel to and from a radiotherapy centre. If the TARGIT-A inclusion criteria were to be used as selection criteria, 54% of patients receiving breast conserving surgery could be offered single dose IORT treatment. Implementing this could save UK patients 2 million miles of journeys and reduce UK CO_2_ emissions by up to 588 tonnes annually (11). Over the past 20 years, TARGIT-IORT has been used in 260 centres worldwide, where around 45,000 patients have been treated. Through this, an estimated 20 million travel miles have been avoided (33).

Currently available cost analyses compare TARGIT-IORT to previous 15+ fraction standard of care (34). The shift to ultra-hypofractionation has undoubtably reduced patient costs, radiotherapy waiting times and allowed more patients timely treatment (35, 36). Radiotherapy department costs are largely fixed and dependent on departmental throughput, therefore the new 5 fraction regimen reduces costs per patient but may not significantly reduce costs if units stay busy. An updated cost benefit analysis comparing TARGIT-IORT to the current standard of care must be elucidated (37, 38).

Vaidya et al. (7) compared the TARGIT-IORT arm against a 15 fraction EBRT regime, contrary to the FAST-forward method introduced as the UK’s standard of care in 2022. Compared to the previous 15 fraction regime, the FAST-forward method reduces the labour, time and financial burden on patients and health systems. The FAST-forward trial reported no statistically significant differences between tumour relapse, survival, normal tissue effects and photographic change in breast appearance, compared to the 15-fraction method (4, 39). Although this may weakly imply that TARGIT-IORT may replicate its efficacy and side effect profile compared to FAST-forward, further studies must be undertaken to compare the two directly. Furthermore, recurrence rate after lumpectomy with the omission of radiotherapy is associated with an increased incidence of local recurrence but no detrimental effect on distal recurrence, therefore TARGIT outcomes should also be compared to a no radiotherapy group (40, 41). Alongside this, authors have scrutinized the TARGIT-A trials, with most criticism describing inadequate data collection, an inappropriately lenient use of the non-inferiority criterion, and focusing data collection from a favourable subgroup of patients (42–46). Current follow up data at 5 years show an increase in local recurrence with TARGIT-IORT, but no overall increase in mortality (7). Further follow up must also be undertaken to properly establish long term efficacy as risk of tumour reoccurrence continues to increase after 7 years (47).

## Conclusion

As healthcare professionals, we have a responsibility to uphold patient care, well-being and quality of life as well as delivering optimal treatments individualised to patients’ needs. More can be done to optimise breast cancer treatments in thousands of patients in the UK. Consideration should be put towards the development and use of IORT in order to improve patients’ quality of life by considering their physical health, mental health and social support before prescribing radiotherapy.

## Data availability statement

The original contributions presented in the study are included in the article/supplementary material. Further inquiries can be directed to the corresponding author.

## Ethics Statement

Written informed consent was obtained from the participant/patient(s) for the publication of this case report.

## Author contributions

MO contributed to the conceptualisation and original draft. SDSM contributed to manuscript review and editing. NC contributed to supervision, conceptualisation, manuscript review and editing. All authors contributed to manuscript revision, read, and approved the submitted version.
